# Dr. Alder's Proofs of the Proximate Causes of Fevers, &c. from Dissections

**Published:** 1809-06

**Authors:** 


### Dr. Alder's *Proofs of the Proximate Causes of Fevers, &c. from Dissections.*

### *In Continuation of his Letters.*

I Come now to the history of the appearances discovered on the dissection of bodies after those diseases which are called fevers, though these diseases are extremely diversified in their appearances and nature, and most other general disorders are in some measure entitled to the same denomination. The term "FEVER," at present, is undefined, and no person has yet been able to say on what the disease implied by it consists. Perhaps Dr. Cullen's definition is as good as any; but there are very many instances and indeed forms of fever which have scarcely a symptom that corresponds with it. Thus many die in the worst fevers in an hour or a few minutes, without any other symptoms than those of syncope, apoplexy, epilepsy, or phrenitis. Many die in the cold stage, before they have had a hot one. Many have no cold stage, (their lungs growing irritated almost as soon as congested) and many have the symptoms which belong to pneumonia, hepatitis, enteritis, dysentery, cholera, &c.; and, as I said before, to syncope, epilepsy, apoplexy, or phrenitis, rather than to what is more strictly called fever. But the truth is, (if my Theory is right) that the proximate cause of fever, when varied a little, is the proximate cause of all these diseases; and all of them might be considered as modifications of fever. And in confirmation of this Theory we shall see, that great congestions of black blood in the veins and viscera cause the bad symptoms in all these diseases; these, however, being sometimes more in one viscus and sometimes more in another, (according to the disease) and sometimes enormous and sometimes small, according to the strength of it and other circumstances.

But I must go a step farther; and say, there is no part of the system which is not exposed to venous congestion and to disease from an impeded transmission through the lungs; and pathological as well as physiological anatomy will support me in this.

I shall begin with those fevers which are the most common, and so proceed to the worst. Morgagni has only about five or six dissections of fever patients, (he having been afraid of contagion) and these are for the most part of people who had died of the intermitting fever. Hoffman has about the same number. Senac has more. Bonetus netus netus has several; these relate principally to the more common forms of fever. Dr. Gregory has given, in his Lectures, so good an abstract of what is found among these writers, that I shall quote his words.

"All notice nearly the same phenomena, viz. inflammations, enlargements, or indurations, in various parts in the abdomen and thorax, suppurations in the omentum or peritoneum; induration and obliteration of the ducts leading to the pancreas; great congestions of black blood in the heart, the large vessels about the lungs, and in the lungs; enlargement of the spleen and liver, and a great redundance of very acrid bile. But sometimes the liver has been found much smaller than usual, and then sometimes of nearly a cream colour; and sometimes the gall-bladder has been found empty. Much oftener, however, it has been found much enlarged, and the vena portæ has been found very wide. Sometimes, and even after the most violent delirium, the head has shewn no *morbid* state, [Dr. Gregory should have said, no *uncommon* state] but *sometimes* [here I would say *much oftener*] it has shewn signs of too great turgescence, or even inflammation, and the lungs too have sometimes shewn signs of previous inflammation."

But it must be remarked, that instead of Hoffman, Dr. Gregory had consulted Cleghorn; and that this circumstance made him dwell much less upon the congestions in the lungs, head and liver, than he otherwise would have done; also made him take more notice of certain secondary affections in the abdomen, which are the effects of a lingering or of a complicated disease; for Dr. Cleghorn's dissections might almost as well be said to be of people who had died after cholera, dysentery, catarrh, or pneumonia, &c. as after a remitting or intermitting fever, his patients having often suffered under all of those complaints, or most of them, in one illness; as we shall see from his own account. "There seems to be *a near alliance* amongst all these diseases. Those who have the essere to a great degree are liable to certain fevers. On the other hand, in the paroxysms of tertians, these cutaneous eruptions are apt to break out. The cholera sometimes has its regular periods like a tertian, as the paroxysms of a tertian are frequently attended with a cholera. Sometimes a tertian is changed into a dysentery, or a dysentery becomes a tertian; and when one of these diseases is suppressed, the other often ensues. Nor is it uncommon for dysenteric fevers to put on the form of tertians, and for the the fits of tertians to be regularly accompanied by gripes and stools." Chap. ii.

Again, "The hooping-coughs, catarrhs, and tertians, during some constitutions of the air, become nearly like pleurisies, or altogether the same; *and the pneumonias came on as agues do*, and had remissions and exacerbations like them, though they, even in this case, required copious bleeding." Chap. vi. However, he distinguishes such complaints as were *most* like strict fevers, and gives the appearances on dissection after them in the following words. "I have examined the bodies of near a hundred who died in these fevers, and constantly found one or other of the adipose parts in the lower belly, the caul, the mesentery, mesocolon, &c. of a dark black complexion, or totally corrupted; the vesica fellea full and turgid, and the stomach and intestines overflowing with bilious matter; the *spleen* large, sometimes weighing four or five pounds, and so exceedingly soft and rotten that it had more the appearance of congealed blood wrapped in a membrane, than of an organical part. In the cavity of the head and breast I saw nothing *extraordinary*, excepting yellow serum."

That the head and lungs were not *remarkably* congested in these fevers was most probably owing to alvine fluxes prevailing so much in them, these, it is well known, usually giving great relief to a congested head, and to congested veins in general. But that such congestions were *often considerable*, is proved *by the effusions of serum* into these parts. Dr. C. dissected "fourteen bodies more, where the lungs had been *principally* affected. In these, I found the lungs were converted into a hard liver-like substance, and sunk in water; and in some of them the sinusses of the dura mater were distended with blood." Chap. vi. He dissected several others, in which dysentery had been the chief complaint. In these he noticed, "the small intestines were sound, the gall-bladder mostly full of dark bile, the *spleen* more or less in a putrid condition; in two, the omentum suppurated, but in all the great guts had mortification in them, chiefly the rectum." But it is evident these latter dissections were made little farther than the supposed seats of these diseases. With respect to the liver and spleen, Dr. Cleghorn further informs us, "that they were frequently found enlarged in this island, not only in men but also in brutes, particularly sheep."

I shall now shew the chief reason why some organs, (especially the liver) are sometimes found smaller than usual usual after fevers, from the work of Doctor Hunter, who quotes the history of twenty-three dissections made by Mr. John M'Colme, and others related by Sir J. Pringle. In these, "the liver was pale and hard in many parts, and in those parts deficient in blood; but where (he adds) it was remarkably so, *there were obstructions in the vena portæ resembling polypi.*" Further, we are assured there was, as might be expected, "in the right ventricle of the heart, the sinus venosus, and vena cava, less blood than common." It is evident these polypi would allow but little blood to pass either into the liver or into the lungs, (that is, of *abdominal* blood) and would of course keep a superabundant quantity in the intestines, &c. Accordingly, we find "the spleen was large and soft, the internal part of the stomach and duodenum reddish or yellow, often blackish, and the other intestines much the same. If the villous coat of the intestines was much affected, there was the *black vomit* found, which, far down, was like *tar.*" No bile, *here,* could be in this black vomit, for almost none was secreted. "The gall found in the gall-bladder being dark and thick, but scanty; the liver pale, and the spleen white." And, indeed, but little if any *could* be secreted, the liver not having any supply by the vena portæ. Hence the black vomit was *chiefly* black blood, which was copiously poured out from all the lower viscera, being unusually congested from their blood not being poured rightly into the vena cava. "The membranes too, as those of the diaphragm, &c. were as if injected. These fevers, like the former, were considerably joined with the dysentery; and the intestines and mesentery in those were found with numerous inflamed tubercles, which, no doubt, these congestions of blood in them would produce. The lungs, though not enlarged, were blackish, and had large livid spots; in these cases they could not be expected to be enlarged. It does not appear that the head was opened.

Dr. Chisholm opened five who had died of the pestilential fever. "The intestines were inflated, inflamed, and sphacelated, particularly the duodenum, a little below the pylorus; the liver was *shrunk* to less than half its natural size, flaccid, and of a yellowish ash colour; the gall-bladder was flaccid and greyish, and contained but a small quantity of ropy bile." All these appearances are precisely the same as in the preceding, only the Dissector does not say he found any polypi in the vena portæ. But this circumstance most Dissectors would overlook, being persuaded that all polypi are formed *in articulo mortis,* or after after death. He then goes on, "The lungs were highly inflamed, and of a livery texture and colour, though no symptoms of a pulmonary affection had been perceived; one of the kidneys was sometimes mortified, though no sign of this had been shewn during life; the coats of the bladder sometimes thickened; much effused blood and serum in the brain; and in one, the blood vessels of the intestines uncommonly turgid." The Author then adds, that "Mr. White opened the bodies of several soldiers, and found the same appearances; and that *similar* appearances were found in twenty bodies which were opened at Brest." According to my judgment, however, they ought not to be called exactly similar, as from the accounts I read, it appears that the large veins, the lungs, the liver, the head, the intestines, &c. were all very much gorged with black blood, and covered with many sphacelated spots; that the gall-bladder often was turgid, and the diseased appearances extended almost all over the interior parts of the body. I do not think too, that the dissections of Rand and Warren agreed so perfectly with those of this respectable author, as he seems to believe, because in one of them the gall-bladder was full of bile, and the ducts were pervious, as well as the liver much enlarged. The lungs also in the other two cases were extremely gorged with black blood, as well as more dense and hard, which they were in all. Dr. Chisholm adds, that Sir J. Pringle found ulcers in the brain in his dissections; and very properly assigns the reasons for it, that Sir John's patients lingered, while Dr. C's did not. In other respects, he says, the appearances were the same in both. From what I have somewhere read, I am disposed to think they were nearly so. I find Sir John particularly notices enlargements, inflammations, &c. in all the viscera; but in his work on the Diseases of the Army, he gives no very particular account of any dissections; and seems surprized at the inflammation of the viscera, and particularly of the brain and lungs.

But there are many dissections related by Dr. Rush, which are as remarkable for having the brain sound, as those of Sir John Pringle and Dr. Chisholm are for having it otherwise. Some reasons, however, may be assigned for this; most of the bodies Dr. Rush alludes to as having nothing unsound (i. e. nothing unusual) in the brain, were well evacuated before death; yet it is remarkable, that it was very customary, in other cases, for the American bodies to discharge "blood from the nose, mouth, and bowels els after death." Doctor Mitchell mentions one case, in which the gall-bladder was distended with black ropy bile, the liver turgid and black, stomach and duodenum full of vessels as if inflamed, the lungs inflated and black, the vena portæ full, and the blood collected in the viscera; *but the vena cava and its branches were empty.* I shall notice this peculiarity afterwards. Dr. Mackittrick says, that in some of the patients who died of the yellow fever, he found the liver sphacelated, the gall-bladder full of black bile, and the veins turgid with black fluid blood. In others the liver no ways enlarged, and its *texture* only vitiated; the stomach, duodenum, and ilium remarkably inflamed in all; the lungs sound. Dr. Hume, in several dead from the yellow fever, found the liver enlarged, and turgid with bile; and in some the stomach and duodenum inflamed. Dr. Lind found in those who died at Cadiz, the liver and lungs of a putrid texture and colour, and the stomach, mesentery, and intestines covered with gangrenous spots. Drs. Phisick and Cathrall found almost every thing sound, except the stomach and beginning of the duodenum; and in two cases very acrid bile. Dr. Annan dissected one body, in which there was unusual turgescence of the brain.

Mons. Assalini says, "On the dissection of several who had died of the yellow fever, or *plague*, at Cadiz in 1800, bilious collections in the liver, the gall bladder distended, the gall ducts obstructed, and in general erysipelatous inflammation over the abdominal viscera, with frequently gangrene of the intestines and stomach, were the appearances noticed."

The Compilers of the Traitè des Causes & de la Peste, have many dissections of those who died of the plague at Marseilles in 1720. Among them, two who died within twenty-four hours from the first attack, and in whom "all the principal parts in the thorax and abdomen were livid, blackish, or of a very deep red; their vessels were filled and gorged with blood of the same colour; an infinite number of those vessels, which in their natural state can scarcely be perceived by reason of their smallness, jumped (sautoient pour ainsi parler*) to the eyes; and those especially which run upon the exterior membranes of the intestines, the stomach, the lungs, and pericardium, were so

* In this it is almost impossible not to recognise the vivid descriptions of a Frenchman.

so sensible, that their minutest ramifications could not be concealed from the sight."

Seven other bodies were opened, of those whose internal congestions did not so suddenly and insidiously extinguish life as those in the last did, but brought on occasionally a little increased action. In them, when the symptoms had been very violent, all the cavities and vessels of the dura and pia mater, and, in fact, all the vessels perceived in the head, were very much distended with thick black blood; the carotid *arteries* were very full; the dura mater was covered with purple spots, and in its posterior part was gangrenous; the lungs were livid, with a gangrenous inflammation on their posterior part, sometimes joined with a whitish colour on their anterior part; the heart was very large, and its cavities were filled with thick coagulated blood; the liver was of double its natural size; the gall-bladder was full of greenish or blackish bile, and the stomach and intestines contained much of the same, mixed probably with blood. Four other bodies were opened, which exhibited much smaller affections of the same parts.

Twelve more bodies were opened, and shewed the same appearances as have just been described; almost always those monstrous congestions, &c. which were found in the first of these bad cases; but often a much greater extension of the congestion, &c. was noticed. Many parts in all the three cavities not before mentioned, were noticed as diseased, in particular the spleen was excessively enlarged, &c.; the stomach, intestines, kidneys, peritoneum, mesentery, &c. all in a similar state with the lungs and liver; the salivary, pancreatic, and mesenteric glands enlarged, and the latter obstructed. Those who had died in the course of twenty-four hours, exhibited as extensive and as great disease as those did who had existed under their fever four, five, or more days. And in all the cases now alluded to, the congestions, inflammations, and gangrenes were the same, whether any marks of reaction or inflammation had appeared during life or not.

Nine other bodies were opened by Mons. Deidier with the same result, except that in two the lungs were not diseased; but in one of these the ventricles of the heart had polypous concretions, and in the other the left auricle was gangrenous. Mons. Rochevalier speaks of a dozen more which were opened; of these, those who had died the most quickly had enlargements in the viscera, but no mortifications; those who lingered, had nothing which the the Dissector calls morbid, that is, they had nothing unusual.

I do not think it is now necessary to mention any more dissections, except seven, which were made by Dr. Chisholm; and these are of a quite different kind: they were of negroes, who inhabited a very cold marsh, and were seized with a fever of the intermitting kind, which annually prevailed among them in that cold marsh about October. The cold stage commonly continued, with great oppression and melancholy, for about *three days!* A hot stage then usually succeeded, and after it an imperfect intermission. Another cold stage then came on, and it increased till the breathing became so difficult that it could scarcely be performed, and the heart then palpitated so that it could be heard! Clammy sweats then soon came on, and the patient died in syncope, seldom getting over this second paroxysm! On dissection, all parts (except in one case) appeared sound, but a large polypous concretion was always found in the right side of the heart, which extended through the whole of the pulmonary artery; and there was a considerable polypus in the left ventricle. In one of these cases the lungs were evidently affected. Dr. Chisholm formed a right judgment of this disease; for, "the moment he perceived its attack, he bled, in order to render *the circulation through the lungs* less difficult and obstructed."

All these cases are fairly taken, and equitably stated; and I have not kept back any article that appeared to be against the conclusions which I make. I intend to offer some Remarks upon them in my next.

*April* 5, 1809.

---

P. S. There was an omission in the printing in the first paragraph of my last letter, implying, that the circumstances there noticed would be adverted to and more fully dilated on afterwards.

*Observations*

